# Recycling of Waste Materials for Asphalt Concrete and Bitumen: A Review

**DOI:** 10.3390/ma13071495

**Published:** 2020-03-25

**Authors:** Md Tareq Rahman, Abbas Mohajerani, Filippo Giustozzi

**Affiliations:** School of Engineering, RMIT University, Melbourne 3000, Australia; abbas.mohajerani@rmit.edu.au (A.M.); filippo.giustozzi@rmit.edu.au (F.G.)

**Keywords:** asphalt concrete, recycling, waste materials, environmental sustainability, advanced materials

## Abstract

Waste management has become an issue of increasing concern worldwide. These products are filling landfills and reducing the amount of livable space. Leachate produced from landfills contaminates the surrounding environment. The conventional incineration process releases toxic airborne fumes into the atmosphere. Researchers are working continuously to explore sustainable ways to manage and recycle waste materials. Recycling and reuse are the most efficient methods in waste management. The pavement industry is one promising sector, as different sorts of waste are being recycled into asphalt concrete and bitumen. This paper provides an overview of some promising waste products like high-density polyethylene, marble quarry waste, building demolition waste, ground tire rubber, cooking oil, palm oil fuel ash, coconut, sisal, cellulose and polyester fiber, starch, plastic bottles, waste glass, waste brick, waste ceramic, waste fly ash, and cigarette butts, and their use in asphalt concrete and bitumen. Many experts have investigated these waste materials and tried to find ways to use this waste for asphalt concrete and bitumen. In this paper, the outcomes from some significant research have been analyzed, and the scope for further investigation is discussed.

## 1. Introduction

A million tons of waste are generated each day around the world. Landfills are used to dump most of this waste. Between 2014 and 2015, Australia produced over 27 million tons of waste [1]. These findings indicate a 6 million ton increase in landfill waste since 2007 [2]. Of the 27 million tons of waste disposed of in 2014–2015, approximately 6.5 million tons were of municipal waste, 13 million tons were of commercial and industrial waste, and 7.1 million tons were of construction and demolition waste [1]. Modern and comfortable lifestyles and innovations in technology, along with industrialization, have increased the quantity and variety of waste being generated, resulting in a severe crisis for proper waste disposal systems [3]. Conventional waste disposal methods are not always efficient and environmentally friendly. Incineration is one popular waste disposal method. However, from research it has been found that the emissions of CO_2_ from incinerators are higher than those for coal, oil, or gas-propelled power plants. Incinerators produce 210 different types of toxic compounds, including mercury, fluorides, sulfuric acid, nitrous oxide, hydrogen chloride, and cadmium [4].

The world’s population is increasing, depleting natural resources. Over recent decades, the retrieval of materials and energy from waste materials has received attention, with the aim of finding a sustainable solution to reduce the exploitation of natural resources and reduce landfill usage, [5]. Sustainability is a thriving field in this millennium [6]. The world is in needs to conserve its resources and determine innovative ways to recycle waste to ensure sustainability [7]. The concept of recycling waste has created a large sector for research. Researchers from various organizations have explored different types of waste materials with green material technologies to reduce environmental impacts and recycle waste in the construction industry [4,8,9,10].

Roads and highways are a critical sector for asset management worldwide. Most highways are flexible in type [11]. Australia has over 350,000 km of surfaced road, and produces over 10 million tons of asphalt concrete per annum [12,13]. Aggregates form up to 95% of asphalt concrete. Therefore, the introduction of alternative aggregates into the production of asphalt concrete and bitumen can help ease the pressure on the world’s landfills and help create sustainable practices for upcoming major road projects around the globe.

## 2. Asphalt Concrete

**Figure 1:**
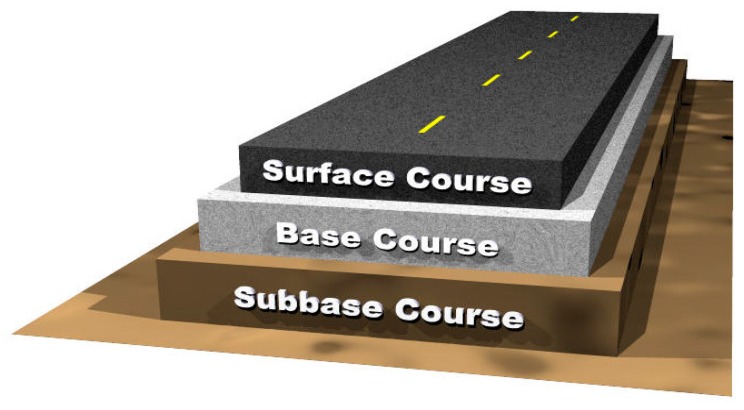
Typical structure of asphalt pavement [15].

Flexible pavement is a widely used type of pavement. Statistics show that 95% of the total highways of the world are made of flexible pavement [7,14]. The type of binder differentiates the two most significant pavement types, which are flexible pavement and rigid pavement. In the case of rigid pavement, Portland cement is used as the binder, and bitumen is used as a binder for flexible pavement. Asphalt concrete is a mixture of aggregates and bitumen. The asphalt concrete mix can be classified into two major categories based on the gradation of the aggregates: hot mix asphalt (HMA) and stone mastic asphalt (SMA). Figure 1 shows the basic structure of a typical asphalt pavement.

### 2.1. Hot Mix Asphalt (HMA)

Hot mix asphalt (HMA) can be dense- or open-graded. As the name suggests, dense-graded HMA has a lower void ratio compared with open-graded HMA. Dense-graded HMA contains a large variety of particle sizes to spread through the asphalt concrete mix effectively. Furthermore, dense-graded HMA suits all traffic condition types and is the most commonly used type of asphalt concrete around the world [16]. Open-graded HMA is typically used in drainage layers due to its higher void ratio, which allows the mix to be more permeable [16,17].

### 2.2. Stone Mastic Asphalt (SMA)

Stone mastic asphalt is a gap-graded HMA, and is commonly used throughout Europe [17]. The aggregates used in SMA mixes are often of higher quality compared with the aggregates used for standard HMA mixes due to their superior physical and mechanical properties, which are required for the stone-to-stone contact structure. SMA’s high content of coarse aggregates creates high rutting resistance and improves the longevity of the structure [18].

### 2.3. Advantages and Disadvantages of Different Type of Asphalt

**Figure 2:**
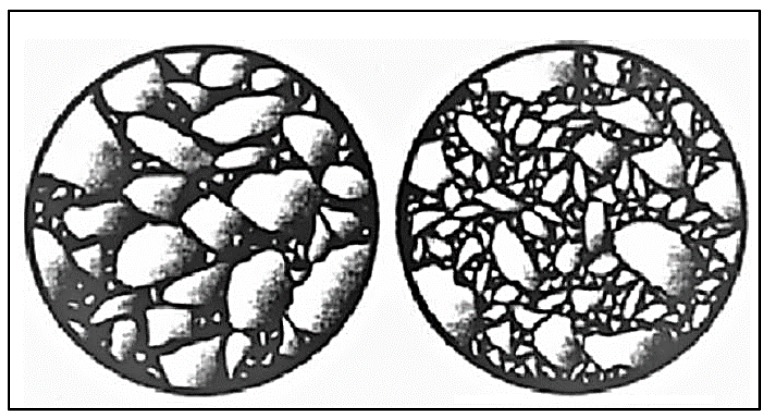
The structural texture of SMA (on the left) and hot dense asphalt (on the right).

A comparative image of HMA and SMA is shown in Table 1. Gradation may vary among different types of asphalt, but the essential ingredients are mostly the same. In the case of HMA, open-graded aggregates and bitumen are used. On the other hand, gap-graded aggregate, fibers, and bitumen are used in SMA [19]. Figure 2 exhibits the structural texture of SMA and HMA.

## 3. Bitumen

**Figure 3:**
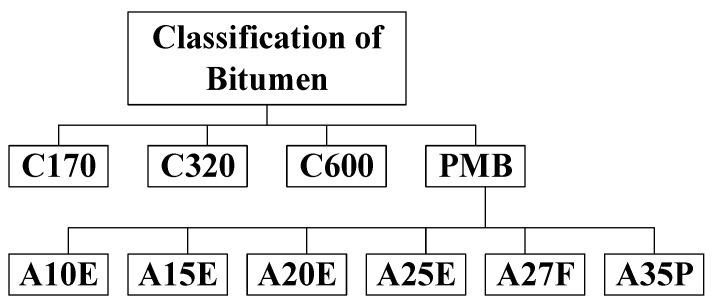
Classification of bitumen according to the Australian Standard for the construction of pavements.

Bitumen is a viscoelastic complex hydrocarbon that is black or brown. Although there are a few natural sources of bitumen available, bitumen is generally sourced from crude oil refineries [20]. Due to its waterproof and viscoelastic nature, bitumen is used as the binder for the construction of flexible pavement all over the world. Bitumen can be classified in three ways: through penetration grade, performance grade, or viscosity. Nowadays, bitumen classification based on viscosity grade is gaining popularity. The available types according to the Australian Standard (with a typical viscosity of bitumen of 60 °C) for the construction of flexible pavements, with the exception of the polymer-modified bitumen (PMB) class), are provided in Figure 3 [21].

Around the world, researchers are working to improve the properties of these materials to ensure sustainability in the pavement construction sector [7,20,22]. The recycling of waste materials for use in asphalt is recognized as a very efficient method, as it improves the pavement quality, and, at the same time, helps to manage and recycle different waste products [7]. Many researchers have investigated the use of different waste materials in bitumen. Plastic and polymer-based modifiers have been used extensively for a long time. Many industries have adopted plastic rubber and polymer-modified bitumen for the construction of roads [22,23,24]. In contrast, many researchers have investigated the use of regular household residues like waste cooking oil in bitumen. In some cases, they have recommended an optimum amount of waste cooking oil in bitumen of up to 5% (by weight) to ensure that any resultant compromise in the performance is minimized [22,25]. Intending to achieve better aging resistance, researchers have used palm oil fuel ash (POFA) to modify bitumen and found that POFA in bitumen can work as a rejuvenator for the binder [22,26,27]. Different types of fiber have been used in construction materials to alleviate the global waste management issue [28]. Several studies have found that fiber can improve the performance of bitumen [28,29,30,31]. Researchers have investigated the use of synthetic fibers like polymer fiber, steel fiber, and carbon fibers in asphalt concrete [28]. It has been found that carbon fiber can improve the electrical properties of asphalt but compromise the mechanical performance of asphalt concrete, while steel fiber improves the stability of asphalt [32,33]. Industry uses cellulose fiber to reduce binder drain-off during the transportation of the mix from the plant to the construction site [34,35]. As cigarette butt filters are made up of cellulose acetate-based fiber, they could represent a potential replacement for the natural cellulose fiber used in stone mastic asphalt. Recycling suitable waste in bitumen in a proper manner is a sustainable way to contribute to solving the worldwide waste management problem [36].

## 4. Use of Waste Materials in Asphalt Mix and Bitumen

Waste materials like plastic, marble quarry waste, building demolition waste, ground tire rubber, waste cooking oil, palm oil fuel ash, coconut, sisal, cellulose, polyester fibers, starch, plastic bottle, waste glass, waste brick, waste ceramic, waste fly ash, and cigarette butts have been reviewed, and methods of recycling in asphalt concrete and bitumen have been discussed. The following sections have been covered in the review of each materials.

(1) Selection of waste material.
(2) Source, characteristics, and common use.
(3) Method of recycling in asphalt concrete and bitumen.
(4) Discussion on the performance of modified asphalt concrete and bitumen prepared with waste materials.

### 4.1. Plastic Waste

Plastic is among the top waste items worldwide. Plastic waste comes in many forms. Common sources of plastic waste are plastic bags, bottles, cups, and straws. Plastic is a polymer-based material which is non-biodegradable. Due to a low manufacturing cost, convenience in carrying and storage, and waterproof nature, plastic has been extensively used around the globe as a household item.

Different types of plastic waste have been used in asphalt as additives. A study was carried out in Turkey to investigate the effect of high-density polyethylene (HDPE) modified binder in hot mix asphalt (HMA). HDPE was mixed with the bitumen content at proportions of 4%–6% and 8% (by weight of optimum bitumen content) [37]. Results of the prepared sample showed increased Marshall stability, Marshall quotient (MQ), and flow. When HDPE-modified binder is used in asphalt mix, resistance against permanent deformation increases, and at the same time, the process helps in recycling plastic waste. Research work in Saudi Arabia has reported that an increased level of industrialization and fast urbanization led to an increase in solid plastic waste. Authors investigated the effect of different types of plastic waste, including HDPE, in asphalt binders. Results showed an increase in resilience modulus, and a model indicated improvement in rutting and fatigue performance [38]. The difference between the various types of polymers like polyethylene, polypropylene, polyvinyl chloride, styrene-butadiene block copolymer, and styrene-isoprene block copolymer relates to the manufacturing process through polymerization. Each type of polymers stand alone in properties like hardness, viscosity, transparency, temperature susceptibility, color, and type of additive used. Table 2 shows the advantages and disadvantages of different types of polymer plastic in the asphalt binder.

An artificial neural network study and as multiple linear regression analysis were carried out, aiming to predict permanent deformation of HDPE-modified asphalt mix. The model showed that up to 7% addition of HDPE waste materials in asphalt mixture reduced the final strain of the mixture and reduced permanent deformation under dynamic loading conditions [40].

Plastic bottles are a ubiquitous household item, and waste plastic is being dumped into landfills every day. Researchers have found that this waste has the potential to be used as a secondary aggregate [41]. The economic issue of polymer-modified asphalt mixture led a team of researchers in Malaysia to determine the effect of incorporating waste plastic bottles into stone mastic asphalt (SMA). Plastic bottles containing polyethylene terephthalate (PET) were mixed with SMA at several differing percentages. The engineering properties of SMA mixed with PET were investigated, and results were statistically analyzed. The results indicated a significant positive effect on the properties of the SMA mix [42]. Recently, another group of research enthusiasts prepared asphalt samples with 1% of waste plastic derived from PET bottles; they concluded their research with success, and they proved that waste bottles could be recycled in the construction of flexible pavement as aggregates [43]. In this research the types of sample were asphalt mixture with 5% glass, 5% plastic, 2.5% glass and 2.5% plastic; 4% plastic and 1% glass; and 1% plastic and 4% glass. Marshall stability and flow results showed an excellent prospects using 5% plastic in asphalt concrete. The adapted results are given in Table 3.

### 4.2. Quarry Waste

**Figure 4:**
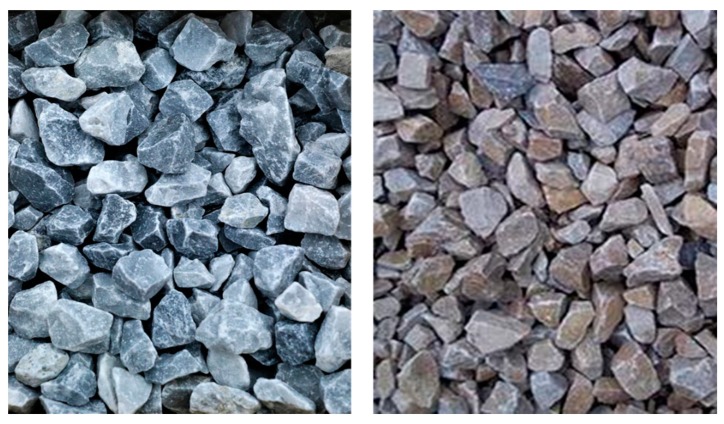
Quarry waste (left) and traditional aggregate (right).

Quarries in different parts of the world are generating large quantities of waste. Mine exploration and extraction of minerals and valuable stones from quarries require digging and blasting, resulting in waste materials and recoverable aggregates. Aggregates from quarries possess very similar properties and appearance to conventional aggregates. In Turkey, industrial waste from marble quarries was proven useful for asphalt pavement by researchers from Afyon Kocatepe Üniversity. Increased demand for aggregate for the asphalt industry and deterioration of the general texture of the Earth’s surface due to the quest for new sources motivated them to use aggregates produced from a marble quarry. During the study, researchers compared aggregates produced as waste from a homogenous marble and andesite quarry with the standard aggregates already in use for the asphalt pavement industry. The results of this research show that the physical properties of the aggregates are similar to the standard aggregates. These aggregates can be used for the construction of asphalt pavement suitable for light to medium traffic conditions [44]. The mining sector produces many waste products. These wastes can be turned into resources by proper innovation and processing methods. Construction of roads and highways require a large amount of aggregates. Conventional granite and basalt aggregates are expensive, and many countries of the world rely on importing these aggregates for their road construction. In India, limestone mining waste was processed and reformed to different sizes according to the gradation table. Asphalt mix samples were prepared by replacing up to 50% of conventional basalt aggregates with the aggregates obtained from mining waste. All the samples fulfilled Marshall design parameters for low-volume roads [45]. Figure 4 shows quarry waste and conventional aggregate for a visual comparison.

### 4.3. Building Demolition Waste

Demolished buildings generate a significant amount of waste each day, as 90% of this waste is disposed of in landfills [46]. A study in Kuwait showed the potential feasibility of demolished building waste for use in aggregates. In this study, Marshall samples were prepared with aggregates obtained from demolition waste. All samples passed the standard requirements based on laboratory investigations. In Spain, researchers evaluated and investigated laboratory and in situ mechanical properties of non-selected recycled aggregates from building demolition waste. They used this waste as an unbound aggregate for the base and sub-base layer of the pavement. Mechanical performances of the road were within acceptable limits [47]. In order to reduce pollution and the burden on landfills, a potential solution could be the recycling of demolition waste for construction material for roads, giving a second life to raw materials [48].

### 4.4. Ground Tire Rubber

**Figure 5:**
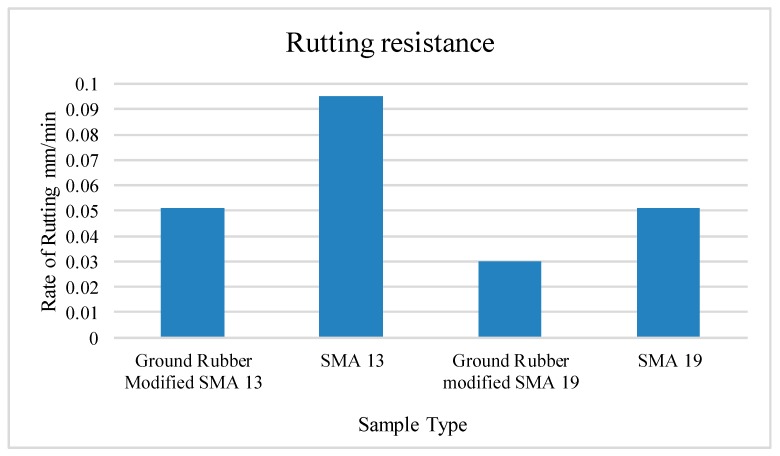
Rate of rutting for SMA modified with ground rubber [49].

Several research works have been carried out to utilize ground tire rubber in asphalt pavements. One significant study used ground tire rubber (GTR) produced in Taiwan in the production of stone mastic asphalt (SMA). When the rubber was used, no fiber was needed to stop drain-down. The results in Figure 5 show that at 60 °C, the rutting resistance of the samples was better than that of conventional SMA mix [49]. SMA samples were prepared with aggregates with a maximum of 13 mm (SMA 13) and maximum of 19 mm (SMA 19). Researchers have also studied ground tire rubber because of the increase in the number tires being dumped into landfills each day [50]. A recent study indicated that the addition of ground tire rubber in asphalt binder enhanced high-temperature properties [51,52]. Pouranian et al. (2020) investigated environmental concerns with respect to the recycling of crumb rubber in bitumen and found that emissions could be reduced with the use of additives in warm mix asphalt (WMA) [53]. Ding et al. (2019) utilized crumb rubber as the rejuvenator for reclaimed asphalt concrete (RAP) and observed improved low-temperature performances [54].

### 4.5. Waste Cooking Oil and Palm Oil Fuel Ash

**Figure 6:**
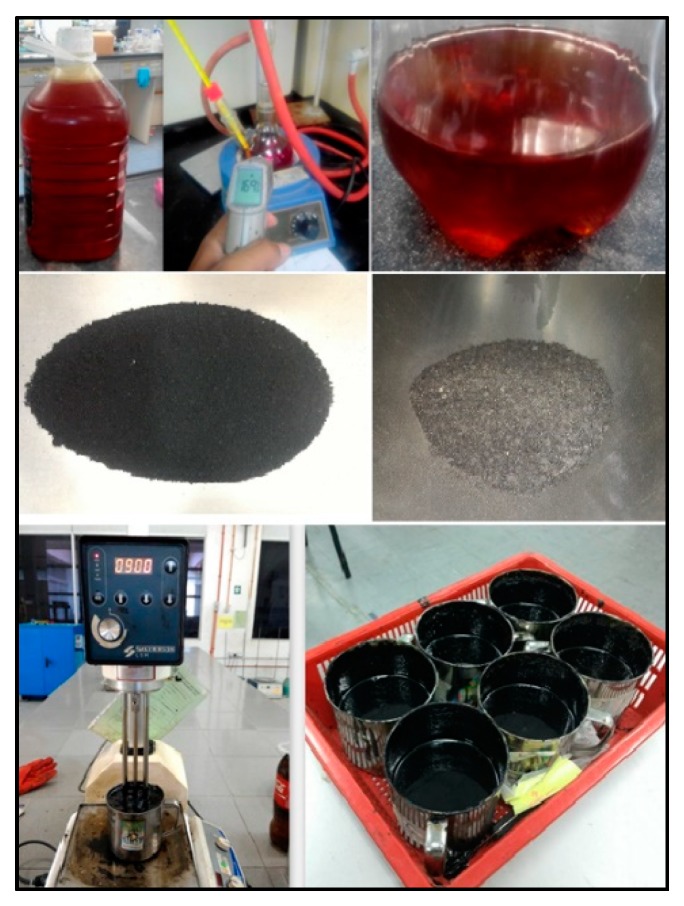
Asphalt binder (bottom right) modified with waste cooking oil (top), crumb rubber (middle left), palm oil fuel ash (middle right), and the blending process (bottom left) [22].

**Figure 7:**
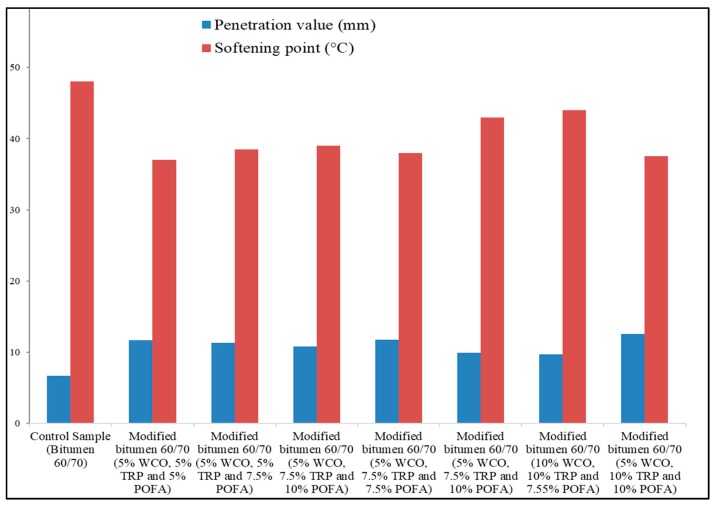
Physical test results of the asphalt modified with waste cooking oil (WCO), tire rubber powder (TRP), and palm oil fuel ash (POFA) [22].

Waste cooking oil is a prevalent type of waste product. Households and restaurants generate a large amount of burnt cooking oil. Hence, the management of used cooking oil is an environmental issue. Wastes like burnt cooking oil and palm oil fuel ash can be used in asphalt mix, according to research carried out in Malaysia. Researchers modified bitumen with waste cooking oil, crumb rubber, and palm oil fuel ash with bitumen 60/70 (penetration grade), and compared the binder with neat bitumen. The selection of the materials and the blending process of the bitumen is shown in Figure 6 [22]. The blending procedure was performed at 120 °C for 2 hours at 900 rpm. The result showed an increase in viscosity of the modified binder and improved penetration and rheological properties, making it a suitable binder for asphalt concrete, as shown in Figure 7. 

### 4.6. Coconut, Sisal, Cellulose, and Polyester Fibers

**Figure 8:**
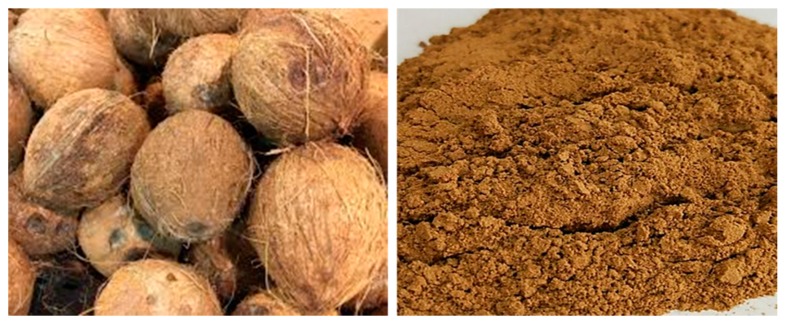
Coconut shell powder (on the right) produced from coconut (on the left), which can be used as fiber.

Coconuts are very common in the tropical regions. Discarded coconut has been recycled for use in many manufactured materials. Coconut shells and fibers have recently been adopted in the asphalt pavement industry. Researchers in Malaysia explored the effect of asphalt mix, where aggregates were replaced by coconut shells. These samples contained coconut shell content of 5%, 10%, and 15% as aggregates. At the same time, coconut fibers were added in the mix, representing 0.3% and 0.5% by weight. Additives were treated by NaOH before the preparation of the asphalt mix to reduce the water absorption property. The result showed better resilient modulus under a temperature of 25 °C when 10% coconut shell aggregate was added [55,56]. In Brazil, coconut, sisal, cellulose, and polyester fibers were used to prepare stone mastic asphalt (SMA) mix. Figure 8 shows the coconut powder, which can be used as fiber for asphalt concrete. In this mixture the amount of bituminous content was higher; hence it was necessary to use fibers to prevent drain-down. Coconut-, sisal-, cellulose-, and polyester fiber-modified SMA mix exhibited high resistance and prevented bitumen from draining down [28,57].

### 4.7. Starch

**Figure 9:**
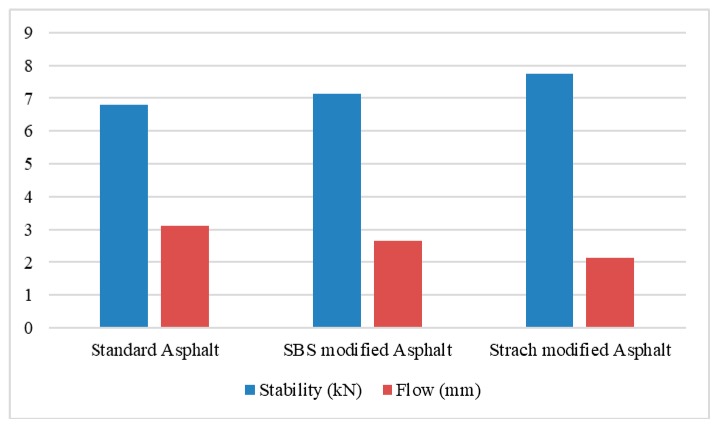
Marshall stability and flow test result of asphalt concrete modified with styrene-butadiene block copolymer (SBS) and starch [58].

Starch (ST) is a natural polymer lighter in weight and is cheaper than other conventional synthetic polymers. Starch can be extracted from trees. Starch was blended with bitumen 70/100 paving grade bitumen. Modified binder was used to prepare stone mastic asphalt with calcium carbonate as a filler; physicochemical, alkali, acid, and fuel resistance tests were performed, as well as the Marshall stability, Marshall quotient, tensile strength, tensile strength ratio, flexural strength, rutting resistance, and resilient modulus tests. The result shows that ST-modified asphalt concrete performs better than conventional and styrene-butadiene block copolymer (SBS)-modified mixture, as shown in Figure 9. Rutting potential and temperature susceptibility can be reduced by the inclusion of ST in the asphalt mixture [58]. 

### 4.8. Waste Glass

Waste glass is generated globally, and mostly comes from glass containers for storage, windows, and windscreens of vehicles. Many research works have been carried out to recycle and reuse waste glass. Glass is a brittle material that mostly contains silica. The inclusion of crushed waste glass from car windscreens in standard asphalt mixtures with less than 5% bitumen resulted in improvements in the mechanical properties in asphalt concrete [59]. The study also found that when the percentage of crushed waste glass was less than or greater than 10%, no further increase to the asphalt concrete’s physical or mechanical properties was seen. Abu Salem and colleagues reported that the addition of waste glass from car windscreens into asphalt concrete had been implemented successfully in the United States since 1990 [60]. It should be noted that a limitation of this study is that fatigue testing was limited to speeds up to 65 km/h, while in real-world scenarios, cars often achieve speeds much higher than 65 km/h on most free-flowing roads. The use of glass cullet as a filler in HMA mixtures has also been studied, and it has been shown that a mix with 6% bitumen content and 15% glass cullet content reduces the strain properties of asphalt concrete at 5°C, 25 °C, and 40 °C compared to the control sample [61].

The incorporation of crushed waste glass into asphalt concrete as a substitute for fine aggregates and a cementitious material with satisfactory results has been recently investigated. The study found that HMA mix properties could be improved with the implementation of crushed glass, even when 20% of fine aggregates were replaced with waste glass [62].

### 4.9. Waste Brick

Waste bricks are generated during the masonry work of construction. This waste brick can be utilized in soil stabilization and can be reused as fillers. The addition of pulverized waste brick as an alternative filler compared to mineral fibers has recently been studied, with positive results. It was apparent that the addition of crushed waste brick as a filler improved the mixture’s mechanical properties at temperatures of 5 °C and 40 °C [63]. At both 5 °C and 40 °C, the mixtures displayed a higher indirect tensile modulus compared to the control sample, which used traditional mineral fillers. The study that investigated pulverized brick waste as a filler also showed promising results regarding the long-term durability of the asphalt, as water sensitivity testing displayed better results in the fatigue life of the concrete asphalt [63,64].

In the state of Victoria, Australia alone, 300,000 tons of waste brick waste were recovered in 2009. However, it was found that reclaimed waste brick may contain up to 30% of other materials, such as concrete or rock [12]. Typical waste brick was found to be suitable in lower quality mixes, and brick with lower porosity levels displayed high compressive strength, allowing a higher quality mix to be manufactured [64]. 

### 4.10. Waste Ceramics

**Figure 10:**
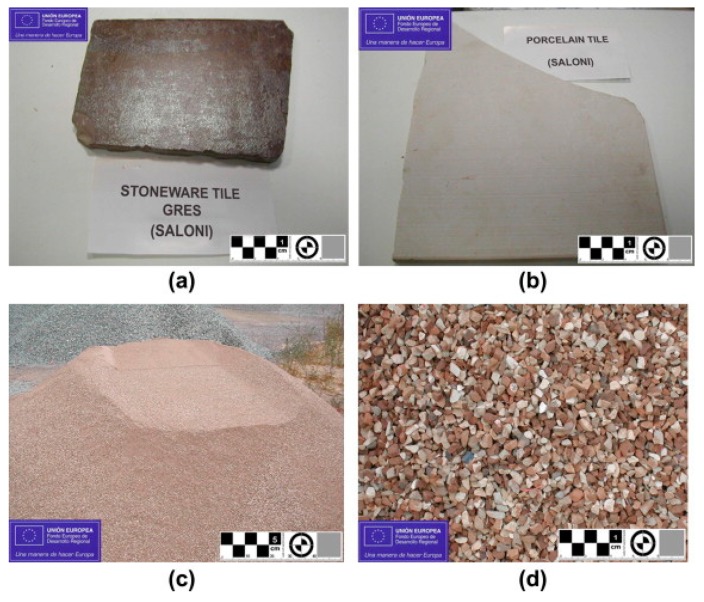
(**a**) Stoneware tile waste; (**b**) porcelain tile waste; (**c**) recycled ceramic aggregates (0–4 mm fine fraction); and (**d**) recycled ceramic aggregates (4–11 mm coarse fraction) [66].

Waste ceramics are mostly generated during the interior work of a building structure. These wastes are thrown into the landfill as part of waste management. Recycling this widely generated heavy waste can reduce the burden on landfills. Pulverized waste ceramic materials have shown promising results for the use of secondary aggregates when used at 20% of the weight of aggregates [65]. The results from the study indicated that the mix that contained more than 20% and less than 100% of the weight of aggregates displayed better mechanical properties compared to the control mix, which utilized limestone as an aggregate. Glazed tiles were excluded in this study, as the glazing applied to the ceramic material disallows proper binding of the HMA mixture. Rutting potential and fatigue testing were also excluded from this study. Research has been conducted to investigate the compatibility of ceramic waste material as a secondary aggregate and it was found that the ideal percentage of waste ceramic was 30% of the weight of aggregates, as shown in Figure 10 [66].

### 4.11. Waste Fly-ash

**Figure 11:**
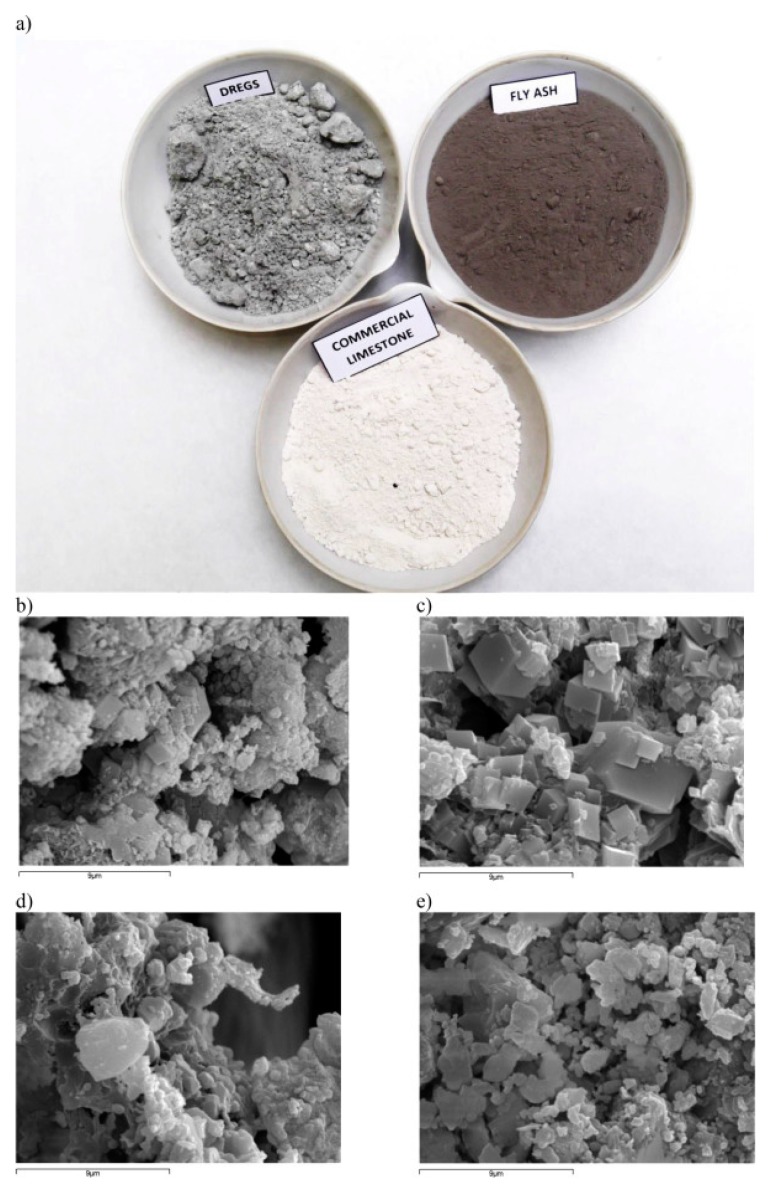
Pictures (**a**) and SEM pictures (**b–e**) of the three fillers: (**a**) dregs, fly ash, and commercial limestone; (**b**) dregs, (**c**) detail of cube-shaped crystals of dregs; (**d**) fly ash; and (**e**) commercial limestone [66].

Fly ash is mostly generated from coal-powered plants as a by-product. Waste fly-ash is one of Australia’s biggest pollutants, with 6 million tons of waste fly-ash was produced in 2014 and 2015 [1]. The use of waste fly-ash generated from the paper industry has been studied in Spain, with less than satisfactory results. Fly ash was utilized as a filler rather than a secondary aggregate in HMA mixtures, resulting in a 7.8% decrease in the resilient modulus, as shown in Figure 11 [67]. Furthermore, the resultant mixture was less stiff and less dense than the control sample. It was suggested that the implementation of fly ash into cold mix asphalt mixtures might provide more satisfactory results due to the fly ash containing hydrated lime. 

However, a recent study by Mohammadinia et al. (2017), considered the suitability of fly-ash as a stabilizer in pavement base applications with positive results. The results displayed that a 15% fly-ash content was optimum for pavement binders [68].

### 4.12. Cigarette Butts (CBs)

**Figure 12:**
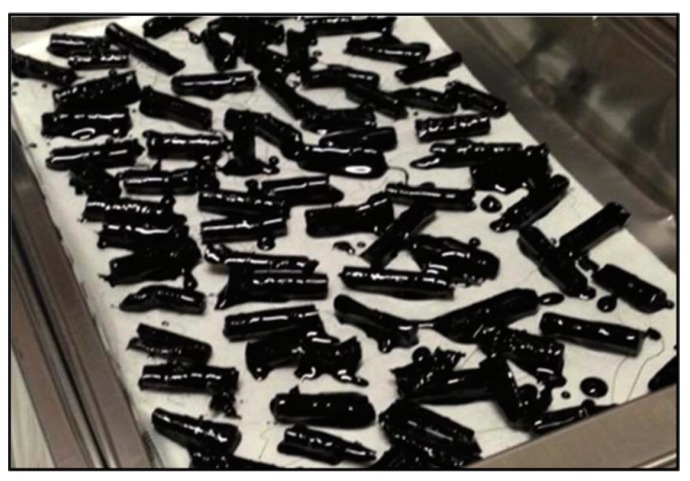
Encapsulated cigarette butts (CBs) used in the research conducted by Mohajerani et al. (2017) [71].

**Figure 13:**
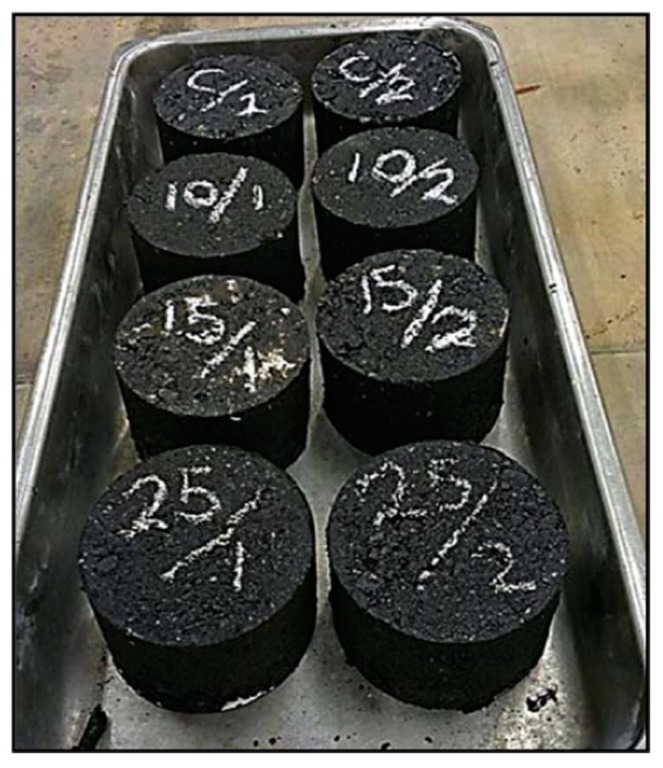
Some CB-modified asphalt samples prepared by Mohajerani et al. (2017) [71].

**Figure 14:**
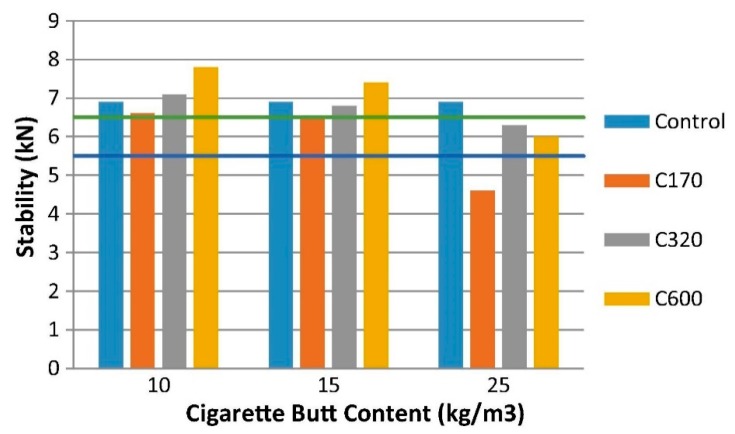
Marshall stability and flow of asphalt prepared with different amounts of CBs (10 kg, 15 kg, and 25 kg CBs in each m^3^ of dense asphalt) encapsulated with bitumen classes C170, C320, and C600 [71].

Cigarette butts (CBs) are the bottom part of a cigarette. They are mostly made of cellulose acetate-based filter and paper [69]. CBs are common form of litter worldwide; hence a sustainable method to recycle CBs could reduce CB pollution problems [70]. Researchers are exploring different ways to recycle this waste to achieve sustainability and reduce the pollution caused by discarded cigarette butts. A recent study at RMIT University initiated by Mohajerani proved that CBs could also be recycled in asphalt concrete. Samples were prepared by the incorporation of encapsulated CBs in asphalt concrete. Figure 12 exhibits the bitumen encapsulated CBs, which were used to prepare asphalt samples. The physical and mechanical performance of the samples was propitious. Furthermore, this work has widened the scope of research for recycling cigarette butts (CBs) in asphalt concrete [71]. Asphalt samples were prepared with incorporation of CBs at 10 kg/m^3^, 15 kg/m^3^, and 25 kg/m^3^, and with no CBs (control samples). Some of the asphalt samples prepared for this breakthrough research are shown in Figure 13. The impact of different quantities of CBs in terms of Marshall stability and flow of asphalt sample where CBs were encapsulated with different classes of bitumen are shown in the Figure 14.

**Figure 15:**
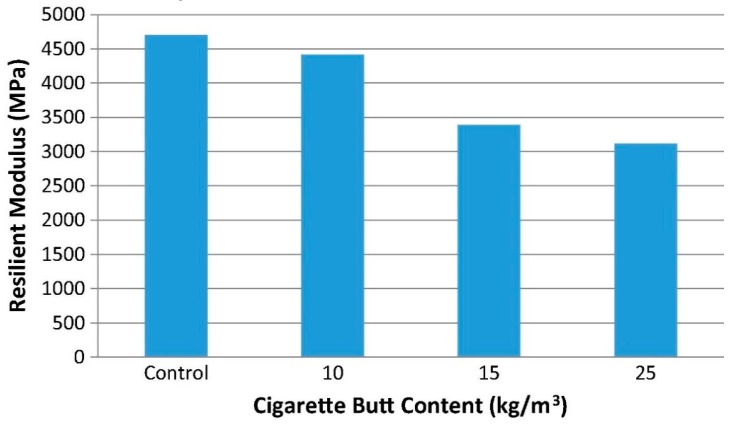
Resilient modulus of asphalt concrete (10 kg, 15 kg, and 25 kg CBs in each m^3^ of dense asphalt) prepared with different amounts of bitumen class C170 encapsulated CBs [71].

Mohajerani et al. (2017) assessed the resilient modulus of asphalt concrete prepared with CBs and found the all the samples met the standard range 2500–4000 MPa for bitumen class C170 [71]. The results are shown in Figure 15.

Recent research showed that cigarette butts (CBs) could be recycled as fiber modifier in bitumen for the construction of asphalt concrete [36]. Different types of bitumen were blended with 0.2%–0.5% CBs as fiber. Results found that CBs as fiber in bitumen enhance the viscosity of the binder and turn the samples less susceptible to temperature change. Resistance to binder drain-off of 0.3% CB fiber-modified bitumen has increased significantly [36]. 

## 5. Significance of Recycling Waste Materials in Asphalt Concrete and Bitumen

### 5.1. Application

Recycling waste materials in asphalt concrete can largely contribute to the sector of waste management. Roads and highways are the world’s largest asset. In the United States, the length of the road network is approximately 6.58 million kilometers [72]. Australia has a road network length of over 832,000 kilometers, and China has a road network measuring more than 4.24 million kilometers [72]. If waste products can be successfully recycled in roads and highways, the global environmental pollution problem due to waste management will be reduced significantly. Asphalt concretes are not only being used in the construction of roads for vehicles but are also being used in the construction of walkways, bike paths, parking lots, and driveways. Bitumen is mostly used as a binder for asphalt concrete. However, bitumen has been used in waterproofing and roofing materials [73,74]. The use of waste materials as an additive can reduce the mixing temperature depending on the devised mixing method. This process can reduce the usage of fuel and minimize emissions [75]. Waste materials can be added to asphalt concrete and bitumen in different forms depending on the type of waste and characteristics. Table 4 summarizes all reviewed materials in asphalt concrete and bitumen. The use of the correct form (e.g., as aggregates, fillers, or modifiers) of waste materials in asphalt concrete and bitumen is important to maintain industry standard performance. 

### 5.2. Economic and Environmental Aspect

Sustainability can be ensured in recycling waste if the final product performs to the same degree or better than the existing product at a low cost and, at the same time, entails some environmental benefit (e.g., low emissions, less landfill use). The use of waste materials in asphalt concrete and bitumen presents a prominent prospect of managing those waste sustainably. Plastic and polymer-based waste products are abandoned everywhere. The use of plastic in asphalt is still in its primary stage. However, the use of polymer-modified bitumen has already gained industry attention. The use of polymer-modified bitumen (PMB) in stone mastic asphalt has become common practice. This paved a way to recycle polymers sustainably and introduced an improved binder for the construction of asphalt concretes. Quarry waste has significant prospects in terms of economic and environmental uses. These waste materials can be utilized as conventional aggregates, which will facilitate efficient management of quarry waste. Both developing and developed cities are generating more building demolition waste every day. Where countries are densely populated and there is a scarcity of livable land areas, minimizing landfill areas can contribute largely to the socio-economic outcome. Utilizing building demolition waste in asphalt concrete significantly reduces the usage of landfills. Ground tire rubber has the potential to be used in asphalt concrete and bitumen. This waste provides a low-cost solution both as an additive and binder modifier. The use of waste cooking oil in asphalt is still in the preliminary research stage. However, this method provides a solution for recycling waste cooking oil in an environmentally friendly manner. Different types of waste fiber can be recycled in asphalt concrete and contribute to sustainable practice. The use of cellulose fiber has become industry practice for the construction of stone mastic asphalt. Waste glass can be used effectively as a filler in asphalt concrete. Crushed glass was successfully utilized during the Tullamarine Freeway widening in Victoria, Australia [76]. Waste ceramic and bricks can be used as aggregates in asphalt concrete. Conventional aggregates are obtained by blasting natural rock, which creates an environmental issue as natural resources are limited. Used of waste in alternative aggregates and modifiers can reduce the use of natural aggregates and give a second life to waste materials. The use of cigarette butts in asphalt concrete is a very recent concept. The successful incorporation of cigarette butts in asphalt at the industry scale can contribute largely to solving global cigarette butt pollution problems.

## 6. Conclusions

The revolution of advanced materials has brought a new dimension to the pavement industry. New methods and procedures have been introduced to ensure the sustainability and efficiency of the roads. Research work is ongoing to investigate the suitability of different types of waste for incorporation as road construction materials. Materials like polymer and plastic have shown increased Marshall stability and flow. In past research, plastic was incorporated in the binder and improved rutting and fatigue performance. Waste from quarries provides a way to replace conventional aggregates for medium traffic conditions. The use of building demolition waste in the base and sub-base layers of asphalt concrete reduced pollution and gave a second life to the materials. Tire rubber powder has been used in many research and improved high-temperature properties. Waste cooking oil along with palm oil fuel ash helped in replacing up to 5% of conventional bitumen binder for asphalt concrete. Fiber-based waste like coconut, sisal, and cellulose prevented drain-down of bitumen and improved resilient modulus. Asphalt binder modified with starch performed better than the binder modified with SBS. Waste glass, bricks, and ceramics have been used as alternative aggregates and exhibited better mechanical properties. Fly ash has been used as filler in asphalt concrete, and the result showed the potential of fly ash into cold mix asphalt mixture. The use of bitumen with encapsulated cigarette butts in asphalt concrete is a new method of managing waste, which will lead to sustainable waste management with improved asphalt concrete.

### Recommendations and Scope for Further Research

Recycling waste products for use in alternative construction materials may not only be a viable answer for the world’s landfill use problem, but also presents a possibility for strengthening the mix design of asphalt concrete and bitumen. Although the use of these materials may initially increase the price of production, it is reasonable to conclude that the cost of recycling materials will reduce and become appropriate once it has become a common industry practice. Utilization of various types of waste in asphalt concrete and bitumen can ensure sustainability and establish innovative recycling procedures. This revolutionary concept can help save the environment from pollution and help in managing waste. All these materials discussed here are part of very recent research work, whereby most of the cases met requirements and exhibited similar behaviors in laboratory investigations as compared to standard asphalt concrete samples. Knowledge in this sector will help future researchers to identify areas for further study and proper guidelines to follow to achieve success. Sustainability and waste management are critical issues. Advances in the waste management sector and turning pollution into the solution will encourage sustainability and innovation in construction materials. Advanced materials will be introduced into the industry, and unique methods of characterization and analysis of the materials will emerge.

## Figures and Tables

**Table 1:** Advantages and disadvantages of HMA and SMA.

Type of Asphalt	Advantages	Disadvantages
**Hot mix asphalt (HMA)**	Low cost Effective in all traffic conditions	Lower rutting resistance Shorter service life Lesser quality aggregates used
**Stone mastic asphalt (SMA)**	Long service life High resistance to deformation Increased fatigue testing life Noise-reductive properties Decreased water spray when raining	Low skid resistance High cost Increased risk of flat spots occurring due to the SMA design procedure

**Table 2:** Characteristics of polymers used to modify asphalt binders [39].

Serial No.	Polymer	Advantages	Disadvantages	Uses
**1.**	**Polyethylene (PE)**	High-temperature resistance Aging resistance High modulusLow cost	Hard to disperse in the bitumen Instability problems High polymer contents are required to achieve better properties No elastic recovery	Industrial uses Few road applications
**2.**	**Polypropylene (PP)**	No important viscosity increases, even though a high number of polymers are necessary (ease of handling and layout) Low penetration Widens the plasticity range and improves the binder’s load resistance	Separation problems No improvement in elasticity or mechanical properties Low thermal fatigue cracking resistance	Isotactic PP is not commercially applied Atactic PP is used for roofing
**3.**	**Polyvinyl chloride (PVC)**	Lower cracking PVC disposal	Acts mostly as filler	Not commercially applied
**4.**	**Styrene-butadiene block copolymer (SBS)**	Higher flexibility at low temperatures Better flow and deformation resistance at high temperatures Strength and very good elasticity Increase in rutting resistance	High cost Reduced penetration resistance High viscosity at layout temperatures Resistance to heat and to oxidation is lower than that of polyolefins (due to the presence of double bonds in the main chain)	Paving and roofing
**5.**	**Styrene-isoprene block copolymer (SIS)**	Higher aging resistance Better asphalt–aggregate adhesiveness Good blend stability, when used in a low proportion	Bitumen suitable for SBS blends Needs bitumen with a high aromatic and a low bituminous content	-

**Table 3:** Marshall stability and flow results of the asphalt prepared with glass and plastic [44].

Sample Type	Waste Materials Used		Marshall Stability (kN)	Flow (mm)
	Glass	Plastic		
Control	0%	0%	13.42	5.64
Glass	5%	0%	6.67	5.92
Plastic	0%	5%	14.66	5.92
Glass + Plastic Type 1	2.5%	2.5%	11.56	5.61
Glass + Plastic Type 2	1%	4%	14.81	6.26
Glass + Plastic Type 3	4%	1%	11.24	4.08

**Table 4:** Summary of the use of waste materials in asphalt concrete and bitumen.

Type of Material	Possible Recycling in Asphalt Concrete	Performance In Asphalt Concrete	Possible Recycling in Bitumen	Performance in Bitumen
Plastic	As aggregate	Improved Marshall stability	As a binder modifier	Improved resistance to permanent deformation
Glass	As aggregate	Reduced Marshall stability	-	-
Quarry waste	As aggregate	Suitable for low-traffic roads	-	-
Building demolition waste	As aggregate	Met standard requirement	-	-
Ground tire rubber	As additive	Improved rutting resistance	As a binder modifier	Improved binder drain of resistance and high-temperature properties
Waste cooking oil	-	-	As a binder modifier	Improved viscosity
Palm oil fuel ash	-	-	As a rejuvenator	Improved penetration property
Coconut and sisal fiber	As aggregate	Improved resilient modulus of asphalt concrete	As a fiber modifier	Improved resistance to binder drain-off
Starch	-	-	As a binder modifier	Reduced rutting potential and temperature susceptibility
Waste brick	As filler	Improved durability and resistance to fatigue	-	-
Waste ceramic	As aggregate	Improved mechanical properties	-	-
Fly ash	As filler	Reduced resilient modulus in hot mix asphalt	-	-
Cigarette butts (CBs)	As aggregate	Encapsulated CBs improved physio-mechanical properties of asphalt concrete	As a fiber modifier	Improved viscosity and resistance to binder drain off
